# Proteomic Analysis Reveals a Novel Function of the Kinase Sat4p in *Saccharomyces cerevisiae* Mitochondria

**DOI:** 10.1371/journal.pone.0103956

**Published:** 2014-08-12

**Authors:** Uta Gey, Cornelia Czupalla, Bernard Hoflack, Udo Krause, Gerhard Rödel

**Affiliations:** 1 Institute of Genetics, Technische Universität Dresden, Dresden, Germany; 2 Biotechnological Center, Technische Universität Dresden, Dresden, Germany; National Institute of Child Health and Human Development, United States of America

## Abstract

The *Saccharomyces cerevisiae* kinase Sat4p has been originally identified as a protein involved in salt tolerance and stabilization of plasma membrane transporters, implicating a cytoplasmic localization. Our study revealed an additional mitochondrial (mt) localization, suggesting a dual function for Sat4p. While no mt related phenotype was observed in the absence of Sat4p, its overexpression resulted in significant changes of a specific mitochondrial subproteome. As shown by a comparative two dimensional difference gel electrophoresis (2D-DIGE) approach combined with mass spectrometry, particularly two groups of proteins were affected: the iron-sulfur containing aconitase-type proteins (Aco1p, Lys4p) and the lipoamide-containing subproteome (Lat1p, Kgd2p and Gcv3p). The lipoylation sites of all three proteins could be assigned by nanoLC-MS/MS to Lys75 (Lat1p), Lys114 (Kgd2p) and Lys102 (Gcv3p), respectively. Sat4p overexpression resulted in accumulation of the delipoylated protein variants and in reduced levels of aconitase-type proteins, accompanied by a decrease in the activities of the respective enzyme complexes. We propose a regulatory role of Sat4p in the late steps of the maturation of a specific subset of mitochondrial iron-sulfur cluster proteins, including Aco1p and lipoate synthase Lip5p. Impairment of the latter enzyme may account for the observed lipoylation defects.

## Introduction

Mitochondria are essential organelles of eukaryotic cells hosting a variety of metabolic pathways, *e.g.* oxidative phosphorylation, β-oxidation of fatty acids and the citric acid cycle. Additionally, mitochondria are the site of anabolic processes such as synthesis of amino acids, lipids and the assembly of iron-sulfur (Fe-S) clusters [1], [2]. Tight regulation is required to balance these metabolic pathways, especially in terms of coordination of the engaged mitochondrial (mt) and extra-mt compartments. Besides the adaptation of gene expression to the environmental conditions, posttranslational modifications of proteins, *e.g.* by phosphorylation, allow a rapid regulation of enzyme activities. While a steadily increasing number of mt phosphoproteins have been identified during the last years in the yeast *Saccharomyces cerevisiae* [3], [4], the number of known kinases (nine) and phosphatases (five) localized to mitochondria is rather small [5], [6], [7], [8], [9]. Additional kinases may reside within mitochondria as suggested by Tomaska by bioinformatics analysis [10].

In our approach we screened *Saccharomyces cerevisiae* mutants, which either lacked or overexpressed genes encoding putative kinases or phosphatases, for phenotypes related to mt dysfunction. Unexpectedly, the serine-threonine kinase Sat4p (also known as Hal4p) emerged as an interesting candidate, since growth on non-fermentable carbon sources of the respective overexpression strain was impaired. In line with this, bioinformatic analysis of the Sat4p protein sequence reveals a remarkable probability for mt localization of 96% and 61% by the programmes MITOPROT [11] and PSORTII [12], respectively. Experimental data on the subcellular localization of Sat4p are, however, missing, and the known functions of the protein imply a cytoplasmic localization. Sat4p was initially identified as a protein involved in salt tolerance, as its deletion causes sensitivity to sodium salts [13], and its overexpression confers increased halotolerance [14]. Further studies suggested a function in the regulation of the Trk1p/Trk2p potassium transporter and/or the stabilization of other plasma membrane transporters [14], [15]. Recently, a role of Sat4p in the control of carbon and nitrogen metabolism was proposed [16] and a regulating function on the transcriptional activator Gln3p was postulated [17].

In this work, we provide evidence that Sat4p has a dual localization both in the cytoplasm and mitochondria. Furthermore we analyze the effect of Sat4p overexpression on the mt proteome by means of two dimensional difference gel electrophoresis (2D-DIGE) and mass spectrometry (MS). Our data show specific alterations in a subset of Fe-S proteins and the lipoamide-containing subproteome, accompanied by impairment of the activities of the respective enzyme complexes. The sites of lipoic acid (LA) attachment in the respective proteins were experimentally identified for the first time. Our data suggest that Sat4p may have a regulatory function in late steps of the maturation of a specific subset of mt Fe-S proteins.

## Materials and Methods

### Strains, media and growth analysis

*Saccharomyces cerevisiae* wild type (WT) strain BY4741 (Accession no. Y00000) and deletion strain Δ*sat4* (*SAT4*::kanMX4, Accession no. Y03488) were obtained from Euroscarf (Frankfurt, Germany). Fusion of *SAT4* with the *cMyc*-tag was achieved by homologous recombination of a cMyc-integration cassette in the corresponding chromosomal locus of strain BY4741 [18], yielding the strain Sat4-cMyc. Replacement of the *SAT4* promoter region was performed by integration of a *TET*-cassette in the strain Sat4-cMyc, resulting in the strain Tet-Sat4. For expression of full-length or N-terminal truncated (ΔN200aa) Sat4p-cMyc under control of the *GAL1* promoter, a respective cassette was integrated downstream of the *SAT4* start codon or in front of basepair position 603 (corresponding to amino acid (aa) 201) of the ORF. Integration cassettes were PCR-amplified from vector pGA2254 (containing nine tandem repeats of cMyc; kind gift of W. Zachariae), vector pCM224 (containing the TetO_2_-promoter, [19]) or vector pFA6a-natMX6-PGAL1 (containing the *GAL1* promoter, [20]), respectively. Yeast transformation was performed according to Gietz and Woods [21].

Yeast minimal media for selection of transformants and full media containing 2% glucose (YPD) or 3% ethanol (YPE) as sole carbon source were prepared as described [22] using media components from FORMEDIUM (Norfolk, UK). TET-resistant transformants were selected on YPD with 0.5 mg/mL G418 (Sigma-Aldrich, St. Louis, MO) and nourseothricin (Werner Bioagents, Jena, Germany) was used in a final concentration of 100 µg/mL.

For growth analysis on plates cells were incubated in liquid YPD overnight, a dilution series from 10^4^ to 10^1^ cells was prepared of each strain and dropped on the respective solid media. Incubation of plates was performed at 30°C for two (YPD) to four (YPD+1 M NaCl, YPE) days.

### Total protein extraction, isolation and treatment of mitochondria

For isolation of total cellular protein or crude mt fractions cells were grown in 30 mL YPE overnight, harvested, washed with dH_2_O, resuspended in 1 mL MTE buffer (650 mM mannitol, 20 mM Tris-HCl pH 7.6, 1 mM EDTA) and disrupted with glass beads for 5 min in a Mixer Mill MM200 (Retsch, Haan, Germany). After centrifugation for 5 min at 3,500× *g* the supernatant was either used as total protein extract or centrifuged again at 12,000× *g* for 10 min for sedimentation of a crude mt fraction.

For localization studies, 2D-DIGE and measurements of enzyme activities mitochondria were enzymatically prepared. To this end, yeast cells were grown in YPE to early stationary phase and mitochondria were isolated and purified by double gradient centrifugation as described by Meisinger *et al.* [23]. Additionally, a salt treatment (1 M NaCl, 0.65 M sorbitol, 10 mM Tris-HCl pH 7.4) was performed for 30 min on ice to remove proteins, which are peripherally associated with mitochondria. For hypotonic shock treatment, 50 µg of mitochondria were resuspended in buffer (10 mM Tris-HCl pH 7.4) containing 100 mM or 650 mM sorbitol, respectively. After incubation for 30 min on ice, mitochondria were sedimented (12,000× *g*, 10 min) and proteins in the supernatant of shocked mitochondria were precipitated using the methanol-chloroform method [24].

In order to prevent proteins from degradation and to maintain their phosphorylation state, a protease inhibitor cocktail (Roche, Mannheim, Germany) and 1 mM 4-(2-aminoethyl) benzenesulfonyl fluoride (AppliChem, Darmstadt, Germany) as well as phosphatase inhibitor cocktails I+II (1∶100, Sigma-Aldrich) were added throughout the preparations.

### SDS-PAGE and Western blot analysis

Preparation of 10% SDS polyacrylamide gels and protein electrophoresis were carried out according to Laemmli [25]. For Western blot analysis proteins were transferred onto a PVDF membrane (Millipore, Billerica, MA), probed with primary antibodies and detected with HRP-conjugated secondary antibodies and the ECL-Plus Kit (GE Healthcare, Little Chalfont, UK). Primary antibodies were directed against cMyc (Roche), Cox2p (cytochrome *c* oxidase subunit 2; Invitrogen, Carlsbad, CA), Aco1p (aconitase; kind gift of R. Lill), Pgk1p (phosphoglycerate kinase; Invitrogen), Dpm1p (dolichol phosphate mannose synthase; Invitrogen), Cit1p (citrate synthase; kind gift of T. Fox), Ccp1p (cytochrome *c* oxidase; kind gift of W. Neupert), Tom40p (subunit of the outer membrane translocase; kind gift of J. Brix) and lipoic acid (LA; Calbiochem).

### 2D-DIGE and image analysis

Labeling of samples with the minimal dyes Cy2, Cy3 and Cy5 (GE Healthcare) was essentially performed as described by the manufacturer. Briefly, 50 µg of highly purified mitochondria from each strain were sedimented (12,000× *g* for 10 min at 4°C), lysed by addition of 10 µL DIGE-thiourea buffer (7 M urea, 2 M thiourea, 4% (w/v) CHAPS, 30 mM Tris-HCl pH 8.5) and labeled with 200 pM of the respective dye for 30 min on ice. Reactions were stopped with 1 µL 10 mM lysine, mixed and 100 µg unlabeled protein of each sample was added to a final volume of 250 µL DIGE-thiourea buffer. Finally, 250 µL IPG buffer (7 M urea, 2 M thiourea, 4% (w/v) CHAPS, 2 mM DTT, 4% (v/v) Pharmalyte pH 3–10 and 0.04% (w/v) bromophenolblue) was added and application to Immobiline DryStrips (24 cm, pH 3–11 NL, GE Healthcare) was performed by *in-gel* rehydration overnight. Isoelectric focussing as well as electrophoresis in the second dimension using 12% gels were done as previously described [5]. All chemicals were from GE Healthcare except for iodoacetamide (Sigma-Aldrich).

Images were acquired with a Typhoon Trio Scanner (GE Healthcare) using the specific excitation/emission wavelengths for Cy2 (488/520 nm), Cy3 (532/580 nm) and Cy5 (633/670 nm). Matching of gels, spot detection, quantification and statistical evaluation were performed using the Delta2D 4.2 software (Decodon, Greifswald, Germany). Ratios of spot intensities (mean normalized volume; p<0.05) are average values from three independent experiments. Total protein staining of gels after fluorescence image analysis was performed with colloidal Coomassie [26], and selected spots were excised from the gel and subjected to mass spectrometry analysis.

### Protein identification by Mass Spectrometry

Processing of excised protein spots from 2D gels for mass spectrometric analysis (*in-gel* reduction and *S*-alkylation, tryptic/chymotryptic digest, peptide extraction) were performed as described previously [27]. MALDI-MS measurements were done using an Ultraflex MALDI-TOF/TOF mass spectrometer (Bruker Daltonics, Bremen, Germany). Spectra processing and peak list generation were performed using flexAnalysis software (version 2.2) with a signal-to-noise threshold of 10 and exclusion of contaminant ion masses as given in table S1. Peptide mass mapping and fragment ion analysis were done using an *in-house* Mascot server version 2.1 (Matrix Sciences Ltd., London, UK) and the following search criteria: (i) taxonomy, S*accharomyces cerevisiae*; (ii) enzyme specificity, trypsin; (iii) mass accuracy, 50 ppm and 0.8 Da for peptide mass fingerprinting and fragment ion analysis, respectively; (iv) fixed and variable modifications, cysteine carbamidomethylation and methionine oxidation, respectively; (v) maximum of one missed cleavage site; and (vi) database, SwissProt 2011_07 (7742 *Saccharomyces cerevisiae* sequences). Proteins were considered as identified if the peptide mass fingerprint exhibited a significant Mascot score (score >58; *p*<0.01).

For nanoLC-MS/MS experiments, extracted peptides were separated on a UltiMate3000 nanoHPLC system (Dionex, Amsterdam, The Netherlands) equipped with a PepMap C18 nano trap column (3 µm, 100 Å, 2 cm×75 µm i.d.) and a PepMap C18 analytical column (3 µm, 100 Å, 15 cm×75 µm i.d.) directly coupled to the nanoelectrospray source (Proxeon, Odense, Denmark) of a LTQ Orbitrap XL mass spectrometer (Thermo Fisher Scientific). Peptides were eluted with an 80 min linear gradient of 5–45% acetonitrile in 0.1% formic acid at 200 nL/min. Mass spectra were acquired in a data-dependent mode with one MS survey scan (resolution of 60,000) in the Orbitrap and MS/MS scans of the eight most intense precursor ions in the LTQ. MS raw files were processed using ProteomeDiscoverer 1.2.0.208 software and searched using Sequest algorithm and the following search criteria: (i) taxonomy, *Saccharomyces cerevisiae*; (ii) enzyme specificity, trypsin or chymotrypsin (spot 8 and 15); (iii) mass accuracy, 10 ppm and 0.8 Da for precursor ion and fragment ion mass tolerance, respectively; (iv) fixed and variable modifications, cysteine carbamidomethylation and methionine oxidation, arginine and glutamine deamidation, and lysine lipoylation, respectively; (v) maximum of two missed cleavage sites; and (vi) database, SwissProt 2011_02 (6583 *Saccharomyces cerevisiae* sequences). False discovery rates were <1% based on matches to reversed sequences in the concatenated target-decoy database. Proteins were considered if at least three peptides were identified.

### Enzyme activity measurements

Photometric measurements were performed in triplicates in 96-well plates in a volume of 200 µL of the respective enzyme buffer containing 20 µg purified mitochondria. Activity of pyruvate dehydrogenase was quantified as previously described [5], except that coenzyme A was added directly to the buffer and the reaction was started with 3 mM sodium pyruvate (Sigma-Aldrich). α-ketoglutarate and isocitrate dehydrogenase activities were measured in the same manner using 3 mM α-ketoglutarate or isocitric acid (Sigma-Aldrich) instead of pyruvate as substrate. Aconitase activity was determined as described by Drapier and Hibbs [28], but without addition of isocitrate dehydrogenase. To exclude that the differences in aconitase activities are due to changes in cellular isocitrate dehydrogenase, the given values result from a quotient of both activity measurements.

Reactions were followed by measuring absorption at 340 nm using an Infinite M200 plate reader (TECAN, Männedorf, Switzerland). The slope in the linear phase of the reactions was used to calculate the activities in comparison to WT.

## Results

### Sat4p is a protein with dual localization in cytoplasm and mitochondria

As outlined in the introduction the reported functions of Sat4p [15], [16], [17] imply a cytoplasmic localization of the protein. However, experimental data on the localization of Sat4p are lacking, and *in silico* analysis predict a high probability for a localization in mitochondria. To investigate the intracellular localization we generated a strain expressing a cMyc-tagged variant of Sat4p and determined its subcellular distribution.

**Figure 1:**
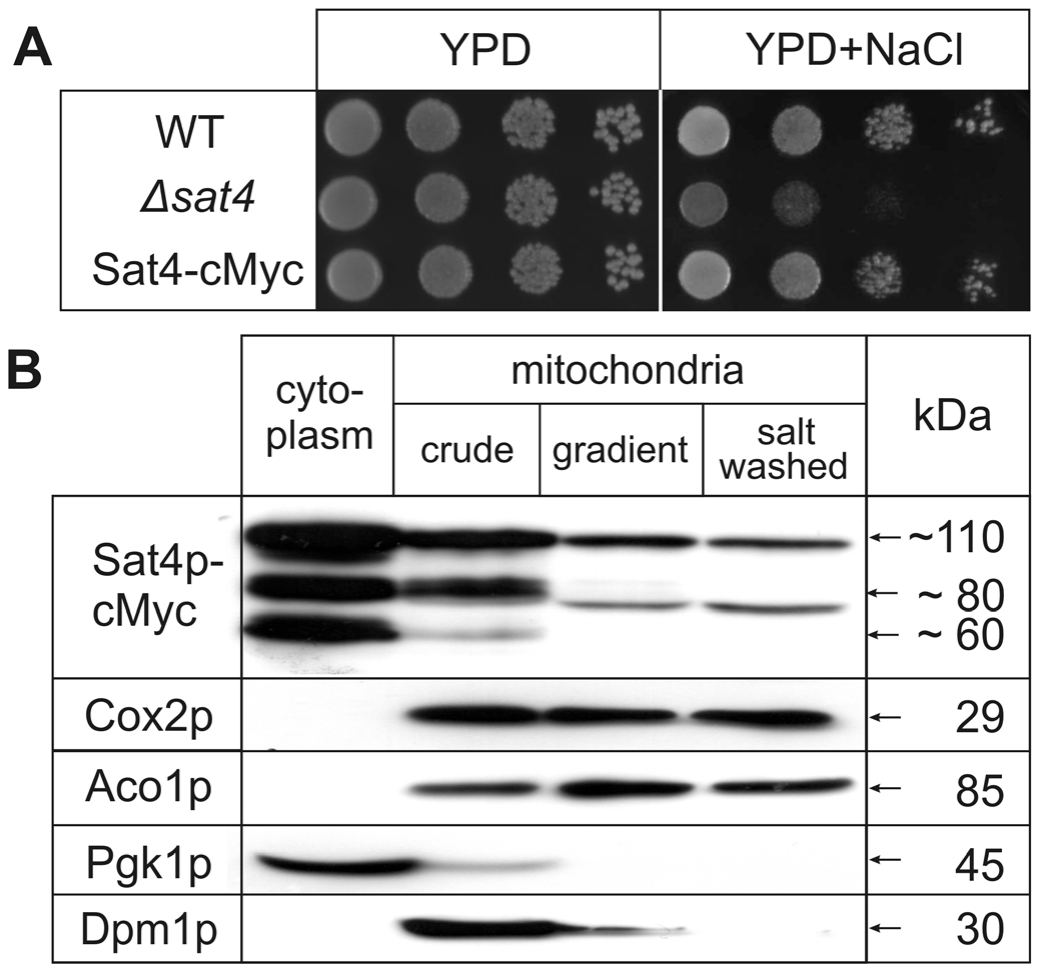
Intracellular localization of Sat4p. (**A**) Functional complementation of the cMyc-tagged Sat4p: Cells of wild type (WT), deletion strain Δ*sat4* and strain Sat4-cMyc were dropped in a dilution series (10^4^ to 10^1^ cells) on YPD with or without 1 M NaCl. Growth was documented after incubation for two (YPD) or four days (+ NaCl) at 30°C. (**B**) Mitochondria of the strain expressing Sat4p-cMyc were enzymatically prepared. 50 µg of cytoplasmic (lane 1) or mitochondrial proteins before (lane 2) and after (lane 3) purification by two successive sucrose gradients followed by a wash step with 1 M NaCl (lane 4) were subjected to 10% SDS-PAGE. Western blot analysis was performed with the indicated antibodies.

We first tested whether the C-terminal cMyc-tag may affect the function of the protein.Yeast cells expressing the fusion protein show a WT-like growth on high-salt medium in contrast to the Δ*sat4* deletion strain (fig. 1A), indicating that the C-terminal epitope tag does not interfere with the protein function. Immunological detection of Sat4p-cMyc (fig. 1B, upper panel) revealed three major signals with similar intensities at sizes of about 60, 80 and 110 kDa in the cytoplasmic fraction. All signals are composed of at least two distinct protein bands, possibly indicating posttranslational modifications. All signals are specific, since they were not detectable in WT mitochondria even after prolonged film exposure (figure S1). Compared to the theoretical molecular weight of about 77 kDa, Sat4p-cMyc exhibits a higher apparent molecular weight possibly due to an altered migration behaviour in SDS-PAGE caused by exceptional features in the protein sequence of Sat4p (see discussion). The bands of about 80 and 60 kDa may represent other conformational or modified forms of Sat4p or may derive from proteolytic degradation, since their intensities varied throughout our experiments.

While the majority of Sat4p is found in the cytoplasm, a smaller, albeit significant, fraction of the protein was detected in the mt fractions (fig. 1B, lane 2–4). Upon purification of mitochondria, the signal pattern changed, and only single signals at ∼110 kDa and ∼80 kDa are visible (fig. 1B). Although *in silico* predictions reveal a high probability for a mt localization of Sat4p, databases like PSORT [12] or the SGD [29] do not clearly predict an N-terminal presequence. The MITOPROT prediction [11] reveals an unusual long prepeptide of 200 aa, which is unlikely to be cleaved off after import. Probably, Sat4p is not (or only partially) N-terminally processed upon import into mitochondria and the upper band (at ∼100 kDa) represents the full-length Sat4p. This assumption may be supported by the migration pattern of the N-terminal truncated (200aa) Sat4p-cMyc (figure S1), which possesses a theoretical molecular weight of 57 kDa, but migrates at an apparent molecular weight of ∼70–75 kDa in SDS-PAGE.

The presence of Sat4p-cMyc in the highly purified mitochondria is not due to contamination by other compartments as indicated by the exclusive detection of mt proteins like Cox2p (inner mt membrane) and Aco1p (mt matrix). Neither proteins characteristic for cytoplasm (Pgk1p) nor for the endoplasmic reticulum (Dpm1p), are detected in this fractions. We furthermore performed hypotonic shock treatments of Sat4-cMyc mitochondria (figure S2). At least part of the mt intermembrane space (as deduced from Ccp1p detection) is released to the supernatant after hypotonic shock, whereas Sat4p-cMyc as well as the matrix protein Aco1p are not detectable in this fraction. These data argue against a localization of Sat4p-cMyc in the mt intermembrane space. However, since outer membrane proteins like Tom40p are present in the shocked mitochondria, it cannot be completely excluded that Sat4p-cMyc is detected in mt fractions due to a firm adherence to the outer mt membrane.

In summary, we conclude from our data that a fraction of Sat4p is localized to mitochondria, either as a component of the mt matrix or tightly adhered to the organelles.

### Overexpression of Sat4p leads to specific changes in the mitochondrial proteome

**Figure 2:**
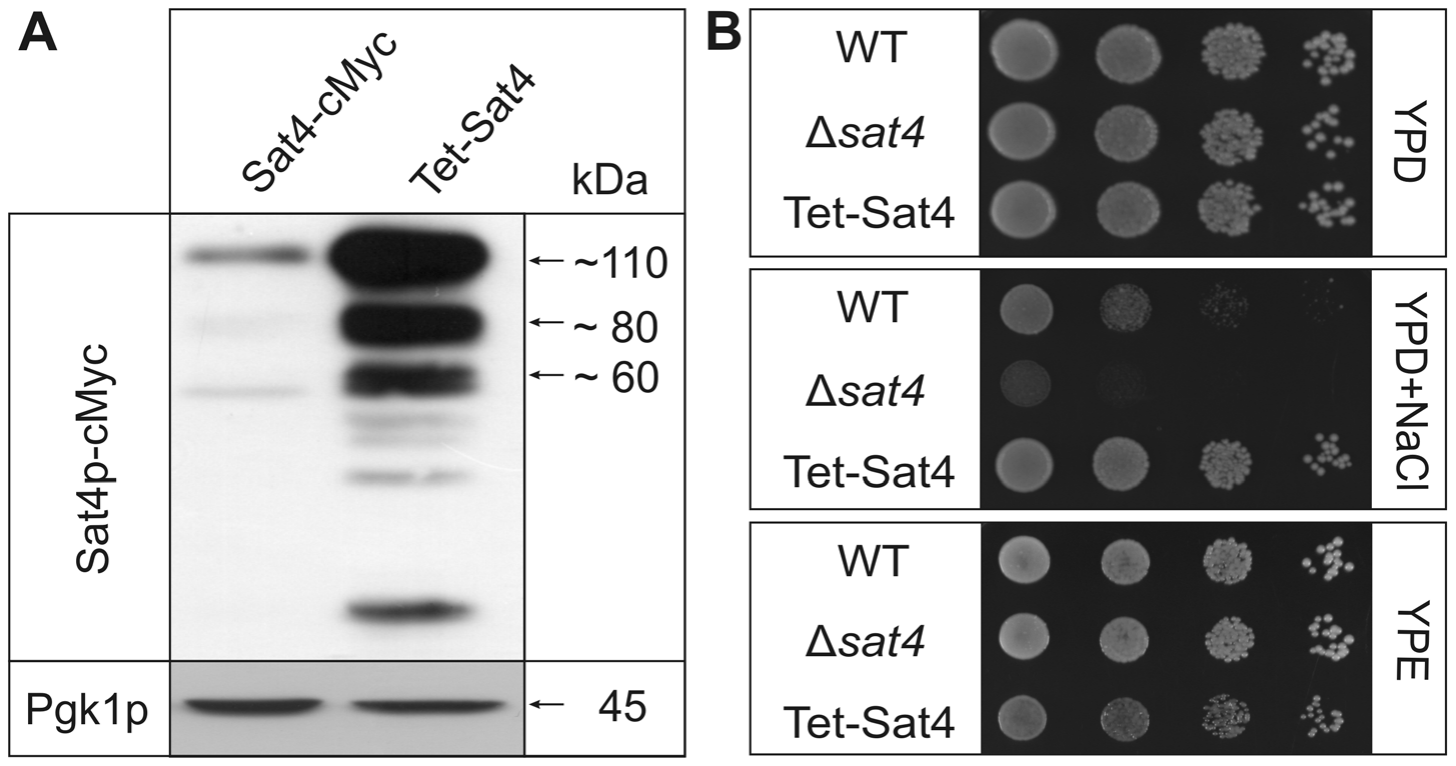
Overexpression of Sat4p. (**A**) For verification of *TET*-promoter driven overexpression of Sat4p, 50 µg of total protein extract from the strains Sat4-cMyc and Tet-Sat4 were separated by 10% SDS-PAGE and subjected to Western blot analysis using a cMyc-antibody. Detection of Pgk1p served as loading control. (**B**) Phenotypes were analyzed by dropping a dilution series (10^4^ to 10^1^ cells) of wild type (WT), Δ*sat4* and Tet-Sat4 on solid YPD, YPD+1M NaCl or YPE. Growth was analyzed after cultivation at 30°C for two (YPD) to four (YPD+NaCl, YPE) days.

The partial mt localization of Sat4p implies an impact on mt functions. To address this question a deletion strain Δ*sat4* and a strain overexpressing *SAT4* (Tet-Sat4) were used. Overexpression was achieved by integrating the strong *TET*-promoter upstream of the *SAT4-cMyc* reading frame. Correct integration was verified by PCR (data not shown) and overexpression was shown by Western blot analysis (fig. 2A). The Sat4p-signals are significantly stronger in the Tet-Sat4 strain (lane 2) compared to the strain expressing the protein under its native promoter (lane 1), and resemble the pattern described above (fig. 1B). In addition, some faint bands in a lower molecular weight range were detected, probably representing degradation products. To compare the phenotypic consequence of *SAT4* overexpression, growth of WT, Δ*sat4* and Tet-Sat4 strains was compared on different media (fig. 2B). On full media containing glucose (YPD) all strains show a comparable growth. Addition of 1 M sodium chloride (YPD+NaCl) led to a reduced growth of the deletion strain, as was reported previously [13], [14]. In contrast, the strain Tet-Sat4 grew better on this medium compared to WT. This observation is in line with a report on a strain overexpressing Sat4p under the control of the *GAL1*-promoter [14]. Interestingly, growth of the Tet-Sat4 strain on non-fermentable carbon sources like ethanol (YPE) was slightly impaired (fig. 2B, lower panel), indicating a disturbance of the respiratory metabolism or mt function. No apparent growth difference was observed between WT and Δ*sat4* on this medium.

**Figure 3:**
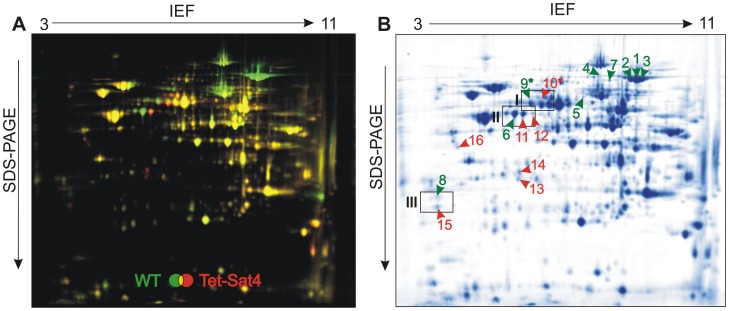
2D-DIGE of mt proteins from wild type and Tet-Sat4 strain. Mitochondria were isolated and purified by sucrose gradient centrifugation. Proteins were labeled with Cy3 (wild type (WT), shown in green) or Cy5 (Tet-Sat4, shown in red) and separated by a non-linear isoelectric focussing (IEF; pH 3–11) followed by 12% SDS-PAGE. An overlay of the channels detected by a Typhoon Trio fluorescence scanner is shown in (**A**). Subsequently, the gel was stained with colloidal Coomassie (**B**). Labeled spots were excised and analyzed by mass spectrometry (results listed in table 1). Protein spots, which showed lower abundance in the Tet-Sat4 strain, are marked by green numbers (1–9), whereas higher abundant proteins are highlighted in red (10–16). Framed gel regions (I–III) are shown in detail in fig. 4.

In order to elucidate the impact of deletion or overexpression of *SAT4* on the mt proteome, purified mitochondria of the strains Δ*sat4*, Tet-Sat4 and WT were analyzed by 2D-DIGE. Mt proteins were labeled with Cy2 (Δ*sat4*), Cy3 (WT) and Cy5 (Tet-Sat4), respectively, and simultaneously separated in a 3–11 NL isoelectric focussing strip (24 cm) followed by a 12% SDS-PAGE. Fluorescence images were subsequently acquired and spot intensities were quantified using the Delta2D software. On the merged image 587 spots were detected. Spot intensities did not differ significantly between WT and the deletion strain Δ*sat4*, which is in agreement with their almost identical growth on YPE. In contrast, the intensities of 16 protein spots of the strain Tet-Sat4 differed more than 2.5-fold (fig. 3). These spots were excised from the gel, and proteins were identified by MS as listed in table 1. Peak lists of spots analyzed by MALDI-TOF-MS or by nanoLC-MS/MS are given in table S2 and S3, respectively.

**Table 1:** Proteins with altered abundance upon overexpression of Sat4p.

Spot	Gene	Swiss Prot No.	Protein	Ratio Tet-Sat4/WT**	MS Method	Mascot Score (PMF)†	Peptides matched	Sequest Score	Peptides sequenced	Sequence Coverage (%)	Predicted		Apparent approx.	
											Mr	pI	Mr	pI
1	ACO1	P19414	Aconitase, mitochondrial	0.2	MALDI-TOF-MS	188	24/52			25	85.7	8.17	73	7.7
2	ACO1	P19414	Aconitase, mitochondrial	0.21	MALDI-TOF-MS	211	22/45			31	85.7	8.17	74	7.3
3	ACO1	P19414	Aconitase, mitochondrial	0.22	MALDI-TOF-MS	105	12/30			19	85.7	8.17	74	8.1
4	SDH1	Q00711	Succinate dehydrogenase [ubiquinone] flavoprotein subunit, mitochondrial	0.26	MALDI-TOF-MS	170	15/44			35	70.8	7.14	76	6.6
5	ICL2	Q12031	Mitochondrial 2-methylisocitrate lyase	0.28	MALDI-TOF-MS	159	20/51			32	65.3	7.21	62	6.2
6	KGD2	P19262	Dihydrolipoyllysine-residue succinyltransferase component of 2-oxoglutarate dehydrogenase complex, mitochondrial	0.33	nanoLC-MS/MS			7132	17	58	50.5	8.88	55	5.4
7	LYS4	P49367	Homoaconitase, mitochondrial	0.39	MALDI-TOF-MS	180	15/25			24	68.0	6.12	74	6.8
8	GCV3	P39726	Glycine cleavage system H protein, mitochondrial	0.39	nanoLC-MS/MS			240	8	56	18.8	4.73	25	4.5
9*	LAT1	P12695	Dihydrolipoyllysine-residue acetyltransferase component of pyruvate dehydrogenase complex, mitochondrial	0.72	nanoLC-MS/MS			2987	22	57	51.8	7.80	59	5.6
10	LAT1	P12695	Dihydrolipoyllysine-residue acetyltransferase component of pyruvate dehydrogenase complex, mitochondrial	2.54	nanoLC-MS/MS			2549	20	52	51.8	7.80	60	5.7
	ALD4	P46367	Potassium-activated aldehyde dehydrogenase, mitochondrial		nanoLC-MS/MS			5337	30	76	56.7	6.74		
	FCJ1	P36112	Formation of crista junctions protein 1		nanoLC-MS/MS			2219	26	58	61.1	6.60		
11	KGD2	P19262	Dihydrolipoyllysine-residue succinyltransferase component of 2-oxoglutarate dehydrogenase complex, mitochondrial	3.62	nanoLC-MS/MS			3659	19	59	50.5	8.88	54	5.5
12	KGD2	P19262	Dihydrolipoyllysine-residue succinyltransferase component of 2-oxoglutarate dehydrogenase complex, mitochondrial	3.65	MALDI-TOF-MS	53‡	5/12			10	50.5	8.88	55	5.6
13	LSP1	Q12230	Sphingolipid long chain base-responsive protein LSP1	3.65	MALDI-TOF-MS	134	11/23			29	38	4.62	27	5.5
14	PIL1	P53252	Sphingolipid long chain base-responsive protein PIL1	3.71	MALDI-TOF-MS	119	11/25			34	38.3	4.54	30	5.5
15	GCV3	P39726	Glycine cleavage system H protein, mitochondrial	3.83	nanoLC-MS/MS			249	5	52	18.8	4.73	22	4.5
16	LSP1	Q12230	Sphingolipid long chain base-responsive protein LSP1	5.52	MALDI-TOF-MS	67	6/12			14	38	4.62	43	4.9

Mitochondrial proteins from WT and Tet-Sat4 strain were subjected to 2D-DIGE analysis (see fig. 3). Significantly altered protein spots (threshold 2.5) were identified by MALDI-TOF-MS or by nanoLC-MS/MS as indicated. PMF, peptide mass fingerprint.

*Abundance change <2.5 fold.

**“Ratio Tet-Sat4/WT” indicates the quotient of intensities (mean normalized volumes) of the corresponding spots from Tet-Sat4 to wild type.

† Mascot PMF scores >58 are significant (p<0.01).

‡ Mascot PMF scores >51 are significant (p<0.05), identification was confirmed by MS/MS of m/z 1745.10.

Among the proteins exhibiting lower abundance upon overexpression of Sat4p, aconitase (Aco1p) showed the most distinct change (up to fivefold, spot 1–3). The Fe-S cluster bearing Aco1p, a major component of the citric acid cycle, is usually detectable in many different migration forms in 2D gels [7], most likely caused by posttranslational modifications. The succinate dehydrogenase subunit Sdh1p (spot 4), another component of the citric acid cycle, was reduced to about a quarter in Tet-Sat4 mitochondria. A similar decrease was observed for the mt isocitrate lyase Icl2p (spot 5), an enzyme of the 2-methylcitrate cycle that is expressed in ethanol-grown cells [30]. A significant decrease in the abundance was further detected for the homoaconitase Lys4p (spot 7), which catalyzes the conversion of homocitrate to homoisocitrate as an essential step in the biosynthesis of lysine [31]. Increased intensities in Tet-Sat4 mitochondria were observed for the protein spots identified as Pil1p (spot 14) and Lsp1p (spot 13 and 16), which are both involved in regulation of the Pkh1p/Pkh2p kinases during heat stress response [32].

The most interesting group of proteins were those represented by more than one spot with opposed intensities in WT and Tet-Sat4: Lat1p (spot 9 and 10), Kgd2p (spot 6, 11 and 12) and Gcv3p (spot 8 and 15). Lat1p, the E2 subunit of the pyruvate dehydrogenase, and Kgd2p, a subunit of the α-ketoglutarate dehydrogenase, are both involved in the citric acid cycle [33]. In strain Tet-Sat4, the more abundant spots of both proteins were horizontally shifted towards a more acidic pI. Although Ald4p (the aldehyde dehydrogenase) and Fcj1p (a mt membrane protein involved in formation of cristae junctions) co-migrate in spot 10 (table 1), its increased abundance is likely to be attributed to an elevated level of Lat1p (see following section). In the case of Gcv3p, we noted a vertical shift of the spots, with the one exhibiting a higher molecular weight showing a decreased intensity (fig. 3B, spot 8 and 15). Gcv3p, the glycine cleavage system H protein, is involved in the glycine catabolic process [34] and required for protein lipoylation [35].

Strikingly, Lat1p, Kgd2p and Gcv3p share one common feature by bearing lipoic acid (LA) as a prosthetic group [35]. To address the aspect of lipoylation, the respective protein spots were analyzed in more detail.

### Lipoylation of mt proteins is affected by overexpression of Sat4p

**Figure 4:**
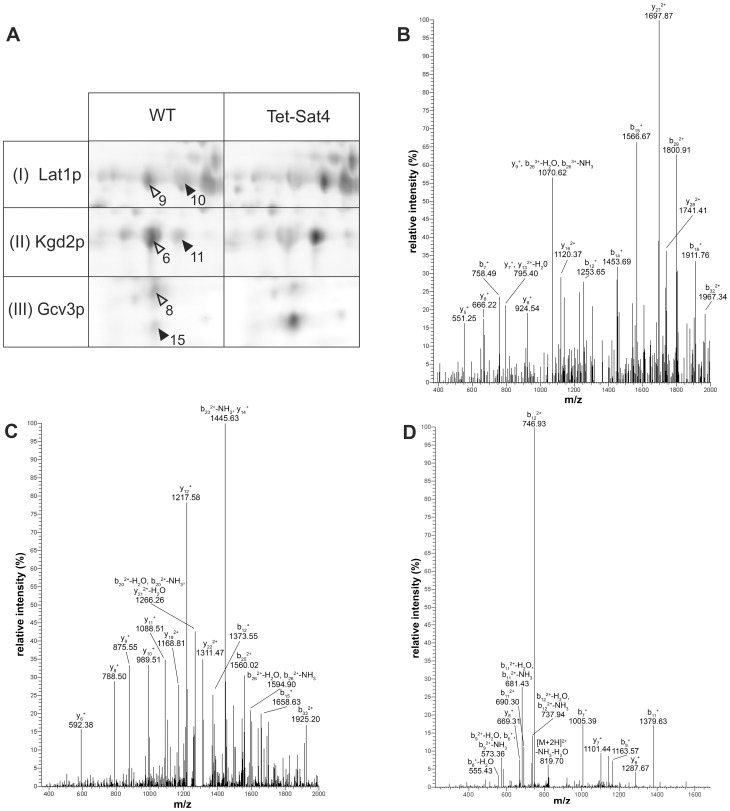
Analysis of lipoylation of Lat1p, Kgd2p and Gcv3p. (**A**) Regions I–III (framed in figure 3) from the 2D gels of wild type (WT) and Tet-Sat4 are shown. Arrowheads indicate spots that are discussed in the text. Spots marked with open arrows represent the respective lipoylated protein form. (**B**)–(**D**) show the MS/MS spectra of peptides used for determination of the lipoylation sites. Triply charged m/z 1384.299 of Lat1p (**B**) and m/z 1332.003 of Kgd2p (**C**) as well as doubly charged m/z 837.392 of Gcv3p (**D**) were analyzed. The Lat1p peptide ^57^KEGDQLSPGEVIAEIETDKAQMDFEFQEDGYLAK^90^ is oxidized at Met78 and carries the LA modification at Lys75. The Kgd2p peptide ^96^NVGDFIKEDELLATIETDKIDIEVNSPVSGTVTK^129^ is lipoylated at the Lys114 residue. The Gcv3p peptide ^96^GSIESVKSASEIY^108^ carries LA at Lys102.

In WT mitochondria, Lat1p and Kgd2p are present in several spots, with the majority of the protein in spots at a lower pH value (fig. 4A, I+II, open arrows) and a minor portion of the protein being shifted towards a basic pI (fig. 4A, I+II, filled arrows). Upon overexpression of Sat4p an increase in the intensity of the basic spots of both proteins at the expense of the more acidic spots is observed. In the case of Gcv3p we observed an intensity shift towards the spot of the lower molecular weight (fig. 4A, III).

We reasoned that the observed alteration in the migration of proteins might result from differences in their modification with LA. Protein spots of Lat1p (spot 9 and 10), Kgd2p (spot 6 and 11) and Gcv3p (spot 8 and 15) were subjected to a detailed MS/MS analysis. In samples of spots 6, 8 and 9 (fig. 4A, open arrows) unassigned peaks in the MS spectra were found, resulting from a mass shift of 304.092 Da. This mass corresponds with LA modified by carbamidomethylation due to iodoacetamide treatment. In contrast, such peaks were not detectable in spot 10, 11 and 15 (fig. 4A, filled arrows), indicating that these proteins lack LA modification.

In the case of Lat1p two putative lipoylated peptides were detected in spot 9 (corresponding to aa 57–90 and 58–90, respectively; table S4). Both peptides were not cleaved at the internal Lys75, as was the case in the MS/MS analysis of spot 10 (table S3). Lipoylation of Lat1p in spot 9 was confirmed by fragment ion analysis of triply charged m/z 1384.299 (fig. 4B) that revealed the sequence ^57^KEGDQLSPGEVIAEIETD**K**AQMDFEFQEDGYLAK^90^ being oxidized at Met78 and carrying the LA modification at Lys75. This was further supported by the MS/MS spectrum of m/z 1341.602 (figure S3). These data provide the first MS-based proof of lipoylation at Lys75, which is located within the predicted lipoyl-binding domain of Lat1p (aa 35–109; [36]).

For Kgd2p, one lipoylated peptide in spot 6 was detected (aa 96–129; table S4) with missed cleavage sites at Lys102 and Lys114. The detection of a non-modified peptide consisting of aa 96–102 indicates Lys114 as the target of LA modification. MS analysis of spot 11 exclusively revealed non-modified peptides of the potential lipoyl-binding domain of Kgd2p (aa 74–147; [37]) (see table S3). MS/MS spectra of triply charged m/z 1332.003 (fig. 4C) as well as m/z 999.252 carrying four charges (table S4) were acquired. Spectra analysis unambiguously assigned the lipoylation within the sequence ^96^NVGDFIKEDELLATIETD**K**IDIEVNSPVSGTVTK^129^ to Lys114.

For the MS/MS analysis of Gcv3p (spots 8 and 15) the respective protein spots were treated with chymotrypsin instead of trypsin for analysis, since the Gcv3p sequence contains long stretches lacking trypsin cleavage sites. Three putative lipoylated peptides were detected in spot 8 (corresponding to aa 96–108 and 80–108, respectively; table S4). Lipoylation of Gcv3p was confirmed by fragment ion analysis of doubly charged m/z 837.392 (fig. 4D) that revealed the sequence ^96^GSIESV**K**SASEIY^108^ carrying the LA modification at Lys102. This site is located within the predicted lipoyl-binding domain (aa 99–113; [34]) and was here identified by a MS approach. Interestingly, the observed spot shift upon overexpression of Sat4p was not horizontally, as detected for Lat1p and Kgd2p, but the spots differed in their molecular weight. One explanation for this observation might be the very acidic nature of Gcv3p and therefore an unusual migration behavior of the different protein forms on 2D gels, *e.g.* by altered SDS binding [38].

As noted above, the intensities of those spots containing the lipoylated protein forms of Lat1p, Kgd2p and Gcv3p were strongly reduced in strain Tet-Sat4, whereby the delipoylated forms of all three mt proteins accumulate.

### Proteomic changes upon overexpression of Sat4p result in diminished activity of the affected protein complexes

**Figure 5:**
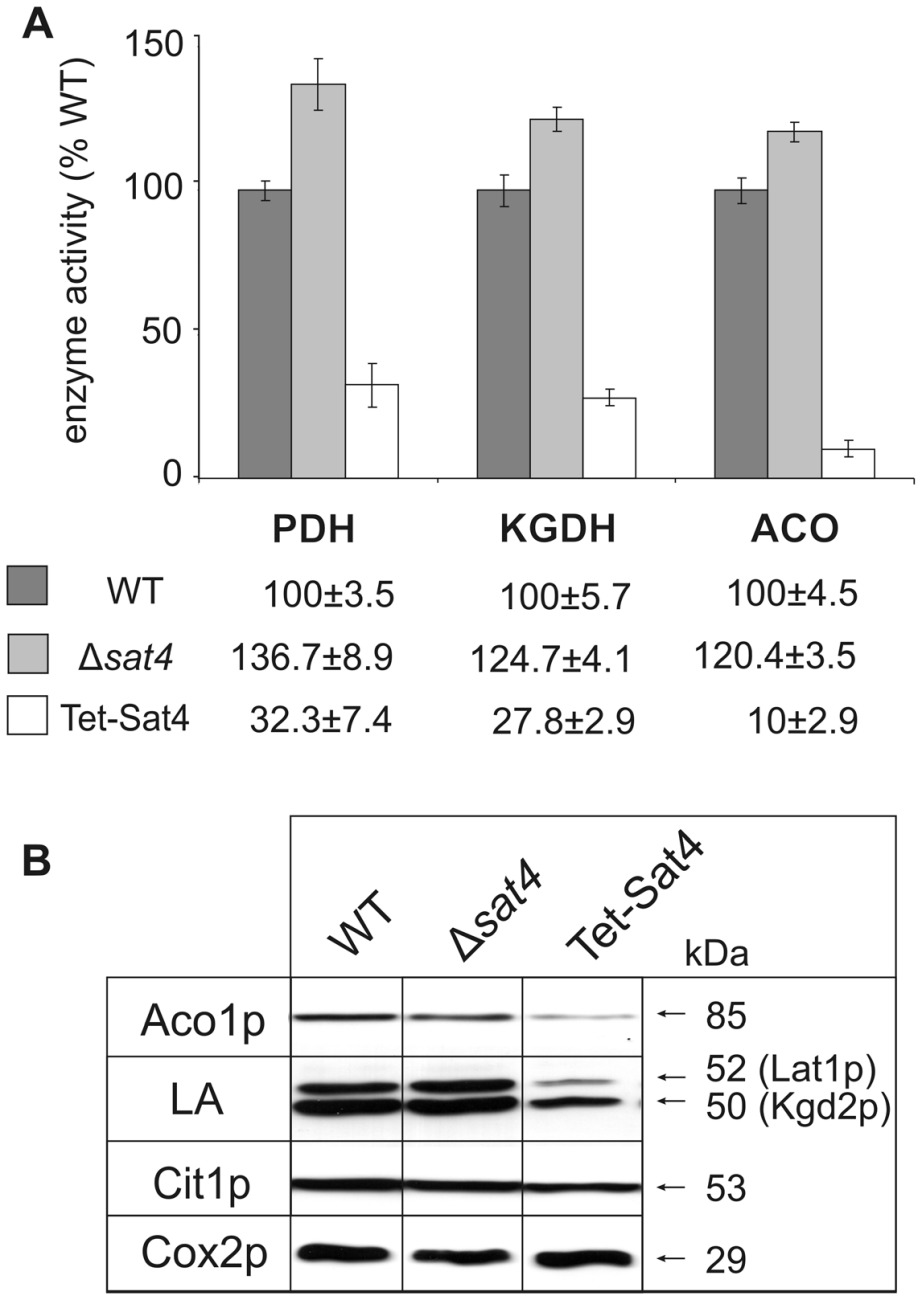
Effect of *SAT4* expression on mt enzyme activities and on steady state concentrations of aconitase and LA-containing proteins. (**A**) Activities of pyruvate dehydrogenase (PDH), α-ketoglutarate dehydrogenase (KGDH) and aconitase (ACO) in purified mitochondria of wild type (WT; dark grey), Δ*sat4* (light grey) and Tet-Sat4 (white) were determined as described in the material and methods section. Mean values derive from three independent measurements (p<0.05, +/− standard deviation) and WT activity was set to 100%. (**B**) Steady state concentrations of aconitase as well as of LA-containing proteins were assessed by performing a 10% - SDS-PAGE of 50 µg mt proteins from WT, Δ*sat4* and Tet-Sat4. Antibodies used for immunodetection were directed against aconitase (Aco1p), lipoic acid (LA), citrate synthase (Cit1p) and subunit II of cytochrome *c* oxidase (Cox2p), respectively.

Next we analysed whether the observed proteomic alterations of Lat1p, Kgd2p and Aco1p are accompanied by changes in the enzymatic activity of the respective complexes. Activities of the Lat1p-containing pyruvate dehydrogenase, of the α-ketoglutarate dehydrogenase bearing the Kgd2p subunit, and of aconitase were measured by photometric assays (fig. 5A). Compared to WT, activities of pyruvate dehydrogenase and α-ketoglutarate dehydrogenase were significantly reduced by approximately 70% in the strain overexpressing Sat4p. These results are consistent with the reduced amount of enzymatically active isoforms bearing the essential cofactor LA. Furthermore, the diminished Aco1p level observed in 2D gels (fig. 3/table 1) correlates with a reduction of aconitase activity (∼10% of WT) in Tet-Sat4 mitochondria. Surprisingly, the activities of the above mentioned enzymes were slightly increased by 20 to 30% in the absence of Sat4p (Δ*sat4*) (fig. 5A). This opposite response of enzyme activities in strains lacking or overexpressing *SAT4* supports the idea of a regulatory function of Sat4p.

Western blot analysis and immunological detection using antibodies directed against aconitase or LA (fig. 5B) confirmed the proteomic data. In line with the results of the 2D analysis, deletion of *SAT4* had no significant impact on the steady state concentration of the detected proteins (lane 2). In contrast, overexpression of Sat4p resulted in reduced levels of Aco1p (fig. 5B, lane 3), while the concentrations of control proteins of the mt matrix (Cit1p) and the mt membrane (Cox2p) remained unaffected. Furthermore, the two bands detected by the LA antibody showed a lower intensity in the Tet-Sat4 strain. Based on comparison with published data using the same LA-antibody [38] the immune reactive proteins are likely to represent the lipoylated forms of Lat1p (upper band) and Kgd2p (lower band).

## Discussion

The serine-threonine protein kinase Sat4p was so far mainly discussed as a factor involved in the regulation of the Trk1p/Trk2p and other plasma membrane transporters [14], [15] and the transcriptional activator Gln3p [17]. However, in line with bioinformatic predictions we show that, although Sat4p resides mainly in the cytoplasmic fraction, a minor portion is localized to mitochondria. When analysed by SDS-PAGE, different migration forms of the protein became apparent pointing towards posttranslational modifications of Sat4p. This idea is supported by *in silico* predictions that indicate several putative modification sites *e.g.* for phosphorylation (Prosite scan; [39]). In line with this, we observed a shift of some signals towards lower molecular weights upon treatment with λ-phosphatase (data not shown). Furthermore, Sat4p contains a remarkable number of 16 cysteine residues and up to eight disulfide bonds are predicted (DiANNA; [40]). Although treatment of SDS-protein samples with different reducing agents had no effect on the protein pattern (figure S4), an incomplete reduction prior to gel electrophoresis or re-oxidation during gel runs, as was reported for other Cys-rich proteins before [41], cannot completely be ruled out.

To investigate the impact of Sat4p on mitochondria, we used both a deletion strain as well as an overexpression strain by placing *SAT4* expression under the control of the very strong *TET*-promoter [19]. Such a dual approach is especially appropriate to investigate the functions of regulatory proteins like kinases [42], since they modify their target proteins transiently. Indeed, effects of the deletion of Sat4p (Δ*sat4*) were comparatively mild regarding mt functions, as significant changes in the growth on non-fermentable carbon sources or in the mt proteome could not be observed. This may indicate the existence of a further kinase with redundant function or a negligible relevance of Sat4p activity under the chosen respiratory cultivation conditions. By contrast, the overexpression strain (Tet-Sat4) is impaired in its growth on non-fermentable carbon sources, accompanied by significant changes in a specific mt proteome.

Among the mt proteins whose abundance is elevated upon Sat4p overexpression, Pil1p and Lsp1p were identified. These proteins were previously shown to play a role in the regulation of the Pkh1p/Pkh2p protein kinases in cellular heat stress response [32]. It was suggested that they might be involved in the recycling of mt membranes and/or the uptake of nutrients by mitochondria [43]. Pil1p as well as Lsp1p are localized to mitochondria in their phosphorylated state [3], [43]. In line with these data, we detected Lsp1p in the 2D gel in several spots that differed not only in their pI, but also in their apparent molecular weight, indicating additional post-translational modification(s). Lsp1p and Pil1p have been previously shown by affinity capture MS to interact with Sat4p [44]. Thus, both proteins might be potential targets of mt Sat4p. However, secondary effects cannot be excluded, considering the involvement of Pil1p and Lsp1p in stress response.

Interestingly, almost all other proteins that were affected in their abundance by the expression level of *SAT4* contain either an iron-sulfur cluster or LA as prosthetic groups. The lipoamide-containing subproteome (Lat1p, Kgd2p and Gcv3p) exhibited a changed electrophoretic migration pattern rather than differences in their steady state concentrations. In the Tet-Sat4 strain, the abundance of the lipoylated isoforms of these three proteins was decreased at the expense of the respective non-lipoylated forms. Using nano-LC MS/MS we were able to experimentally identify the lipoylation sites of all three proteins. Lat1p (subunit of the pyruvate dehydrogenase complex) carries the LA at Lys75, which coincides with the proposed site based on mutational analysis in yeast [36] and is consistent with data of the respective subunit in other organisms [45]. The lipoylated residue of Kgd2p (subunit of the α-ketoglutarate dehydrogenase complex) could be assigned to Lys114, in line with the alignment to the homologous *Escherichia coli* enzyme [37]. In both cases, the diminished lipoylation upon overexpression of Sat4p is accompanied by a reduced activity of the respective enzyme complexes, which can explain the retarded growth of the Tet-Sat4 strain on non-fermentable carbon sources (YPE). Gcv3p, the third known mt protein bearing LA, is modified at Lys102. This modification site was supposed by comparison to the chicken H-protein [46].

Furthermore we observed a decrease in the steady state concentrations of a specific subset of Fe-S containing proteins. Both, the abundance of homoaconitase Lys4p and the aconitase Aco1p, is considerably decreased. In the case of Aco1p the dramatic reduction of the protein level in the Tet-Sat4 strain resulted in a drop to only 10% residual aconitase activity. Besides its central role in citric acid cycle, Aco1p is also critical for mt DNA maintenance [47]. However, long time cultivation did not show any increased loss of mt DNA compared to WT (data not shown) thus excluding secondary effects due to formation of *rho^0^*- cells. The impairment of the citric acid cycle may also explain the reduced concentration of the succinate dehydrogenase Sdh1p [48]. As several intermediates of this metabolic pathway are also used for anaplerotic reactions for the 2-methylcitrate cycle [30], the same argument might be applied to Icl2p due to limited availability of substrates.

The group of proteins affected upon overexpression of Sat4p closely resembles that impaired in deletion mutants lacking enzymes involved in Fe-S cluster biosynthesis, such as Isa1p/Isa2p or Iba57p [49], [50]. These proteins are essential for the formation of the specific Fe-S clusters on aconitase-type proteins (Aco1p, Lys4p) and radical S-adenosylmethionine enzymes (Lip5p, Bio2p) [51]. Maturation of other mt Fe-S cluster proteins like Rip1p of respiratory chain complex III is apparently not affected, thus *SAT4* overexpression has no general effect on mt Fe-S cluster proteins. The lack of Fe-S cluster formation of aconitase-type proteins may not only affect the enzyme activities, but also lower their half life due to enhanced degradation, which could explain the reduced steady state concentrations of Aco1p and Lys4p.

We propose that the reduced lipoylation of Lat1p, Kgd2p and Gcv3p is likely caused by impairment of Lip5 due to incomplete maturation of this essential enzyme for LA synthesis [35]. As previous studies of the yeast mt proteome using 2D electrophoresis [3], [7] our analysis failed to identify the lipoate synthase Lip5p and the biotin synthase Bio2p. Either the low abundance or the unusual biochemical properties of the proteins (*e.g.* the exceptionally basic pI value of ∼10.3 of in the case of Lip5p) may account for this fact. Not surprisingly, a detectable phenotype as a result of an affected function of Bio2p was not apparent. First, lack of biotin depletion can be rescued by uptake of exogenous biotin from the medium [52], and secondly, the majority of biotinylated proteins is found in the cytosol and thus escaped detection in our approach focussed on mitochondria.

Since our data indicate that Sat4p is localized both in cytosol and in mitochondria (or attached to them), it seems plausible that the mt fraction of Sat4p accounts for the observed proteomic changes. However, a role of the cytosolic localized Sat4p cannot completely be excluded, *e.g.* by phosphorylation of a cytoplasmic target that causes a signal transmission into mitochondria. Alternatively, the observed changes might be caused by alterations on transcription level. In this context it is of interest, that a role of Sat4p in the regulation of the transcriptional activator Gln3p has been recently reported [17]. However, the genes encoding the proteins affected by Sat4p overexpression neither contain the Gln3p binding site in their upstream regulatory regions nor show a co-regulation of expression (*Pat-Match*, *Saccharomyces genome database*, [29]). Therefore the observed effects on the mt proteome are likely to result from an independent activity of Sat4p.

**Figure 6:**
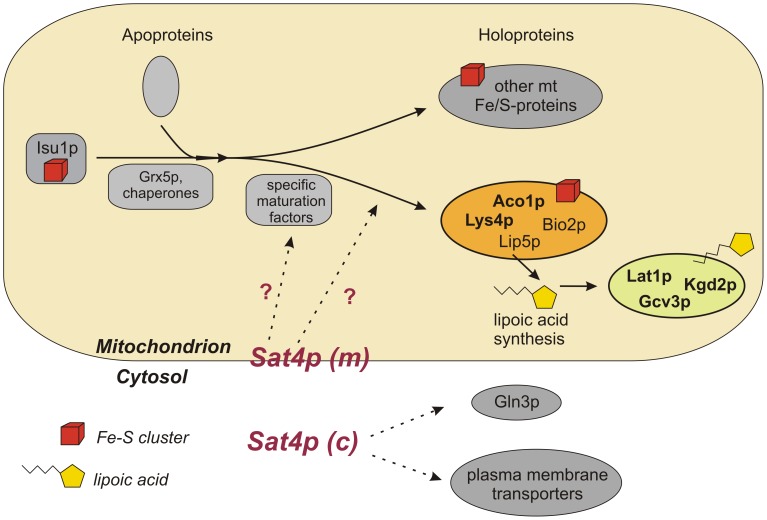
Proposed role of Sat4p in late maturation steps of iron-sulfur cluster proteins in mitochondria. (figure adapted from [51]). The model proposes that the mt fraction of Sat4p (*Sat4p (m)*; matrix localized or tightly adhered to the outer membrane) might negatively regulate late steps in the formation of Fe-S clusters on aconitase-type proteins (Aco1p, Lys4p) and radical S-adenosylmethionine (SAM) enzymes (Lip5p, Bio2p) in mitochondria. The proposed action of mt Sat4p (indicated by question marks) could be either directly or indirectly by modulating the functions of proteins, which are essential in this step of Fe-S cluster attachment [51]. The disturbance of the lipoate synthase Lip5p could explain the impairment of the lipoamide-containing enzyme subunits Lat1p, Kgd2p and Gcv3p. Further putative targets of Sat4p within mitochondria are Pil1p and Lsp1p. The cytosolic fraction of Sat4p (*Sat4p (c)*) might fulfil the reported functions in regulation of plasma membrane transporters [14], [15] and Gln3p [17].

Based on our proteomic data we suggest a model for a novel function of Sat4p in mitochondria (fig. 6). According to this Sat4p acts as a negative regulator in the late maturation step of the aconitase-type subset of Fe-S containing proteins, possibly by directly or indirectly modulating the activities of maturation factors [49], [50]. Pil1p and Lsp1p might be independent targets of Sat4p. Our data show that Sat4p – besides its known functions in the cytoplasm [14], [17] – plays a role in mitochondria. We hypothesize that this function is probably mediated by the Sat4p fraction, which is localized in or tightly attached to mitochondria.

## Supporting Information

Figure S1 **Specificity of cMyc signals (A) and SDS-PAGE analysis of full-length and N-terminal truncated Sat4p-cMyc (B).** (**A**) Mitochondria of the strain Sat4-cMyc as well as wild type (WT) were enzymatically prepared, 50 µg of protein were subjected to a 10% SDS-PAGE and Western blot analysis was performed using cMyc antibodies. Detection of Aco1p and Ccp1p served as loading control. (**B**) 50 µg of total protein extracts of the strain expressing full-length Sat4p-cMyc (left lane) or the N-terminal truncated (200 aa) version of Sat4p-cMyc (right lane) under control of a *GAL1*-promoter were subjected to a 10% SDS-PAGE. Western blot analysis was performed with cMyc antibodies and Coomassie-staining of the gel after blotting served as loading control.
(TIF)

Figure S2 **Hypotonic shock treatment of Sat4p-cMyc mitochondria.** For hypotonic shock treatment of enzymatically prepared mitochondria from the strain Sat4-cMyc, 50 µg of mitochondria per lane were sedimented and incubated for 30 min on ice in buffer containing 650 mM (lane 1) or 100 mM (lane 2) sorbitol, respectively. After centrifugation, proteins in the supernatant of shocked mitochondria (lane 3) were precipitated using the methanol-chloroform method. Samples were loaded to a 10% SDS gel and Western blot analysis was performed successively with the indicated antibodies.
(TIF)

Figure S3 **MS/MS spectrum of triply charged m/z 1341.602 of Lat1p.** The peptide ^58^EGDQLSPGEVIAEIETDKAQMDFEFQEDGYLAK^90^ is oxidized at Met78 and carries the lipoic acid modification at Lys75.
(TIF)

Figure S4 **Influence of different reducing agents on the migration pattern of Sat4p-cMyc.** 50 µg total protein extracts from the strain Sat4-cMyc were treated with different reducing agents. The samples were incubated in 6× Laemmli buffer with 100 mM DTT (lane 1+2), 50 mM TCEP (lane 3) or 5% (v/v) β-mercaptoethanol (β-ME; lane 4) for 10 min at RT. For lane 2, a treatment with 15 mM iodacetamide (IAA) for 10 min at RT followed DTT incubation for alkylation of cysteines to avoid reoxidation. After denaturating samples for 5 min at 95°C, separation was performed in a 10% SDS-PAGE and Western blot analysis was carried out with cMyc and Pgk1p antibodies.
(TIF)

Table S1 **List of contaminant ion masses excluded from processed mass spectra.** Masses were regarded as background and/or calibrant masses.
(XLS)

Table S2 **Peak lists of all spots identified by MALDI-TOF-MS.** Lists were generated as described in the “materials and methods” section. Each spot is also listed in a separate worksheet containing a table summarizing features of detected peaks (m/z, signal-to noise value, quality factor, resolution, intensity, area) and a table listing matched peptides. Identification of spot 12 was confirmed by MS/MS of m/z 1745.10, according details are given in the worksheet.
(XLS)

Table S3 **Proteins identified by nanoLC-MS/MS.** Accession numbers, sequence coverage, number of unique peptides, scores, and lists of identified sequences are listed.
(XLS)

Table S4 **Peptide sequences of post-translationally lipoylated peptides.** Corresponding MS/MS spectra are shown in Figure 4 and Figure S3.
(XLS)
