# Supplementary figures and images for: Proteomic Analysis Reveals a Novel Function of the Kinase Sat4p in Saccharomyces cerevisiae Mitochondria
Source: PLoS One. 2014 Aug 12;9(8):e103956. doi: 10.1371/journal.pone.0103956 (PMC4138037; doi:10.1371/journal.pone.0103956)

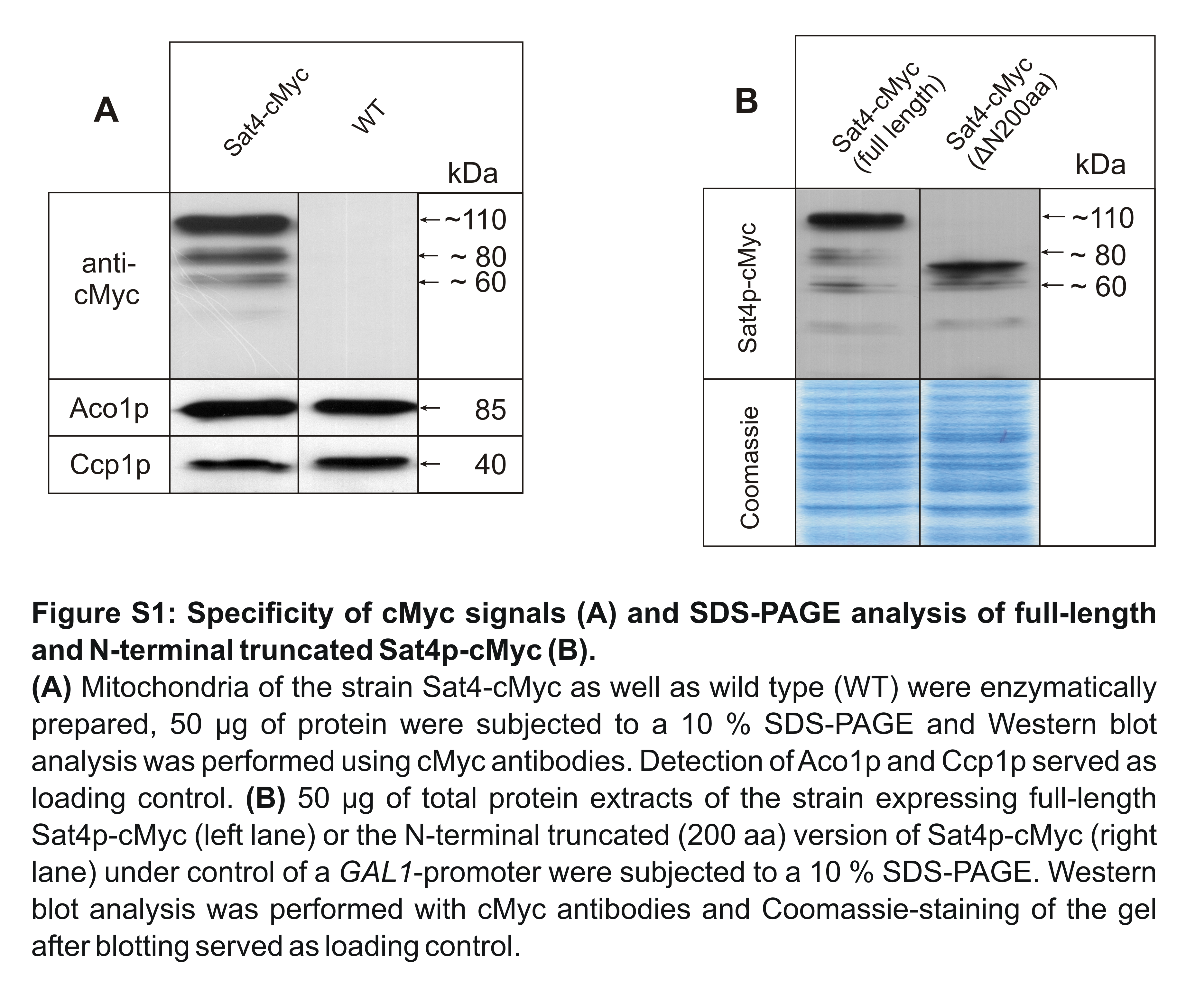

Supplement: Figure S1 — Specificity of cMyc signals (A) and SDS-PAGE analysis of full-length and N-terminal truncated Sat4p-cMyc (B). (A) Mitochondria of the strain Sat4-cMyc as well as wild type (WT) were enzymatically prepared, 50 µg of protein were subjected to a 10% SDS-PAGE and Western blot analysis was performed using cMyc antibodies. Detection of Aco1p and Ccp1p served as loading control. (B) 50 µg of total protein extracts of the strain expressing full-length Sat4p-cMyc (left lane) or the N-terminal truncated (200 aa) version of Sat4p-cMyc (right lane) under control of a GAL1-promoter were subjected to a 10% SDS-PAGE. Western blot analysis was performed with cMyc antibodies and Coomassie-staining of the gel after blotting served as loading control. (TIF) [file pone.0103956.s001.tif]

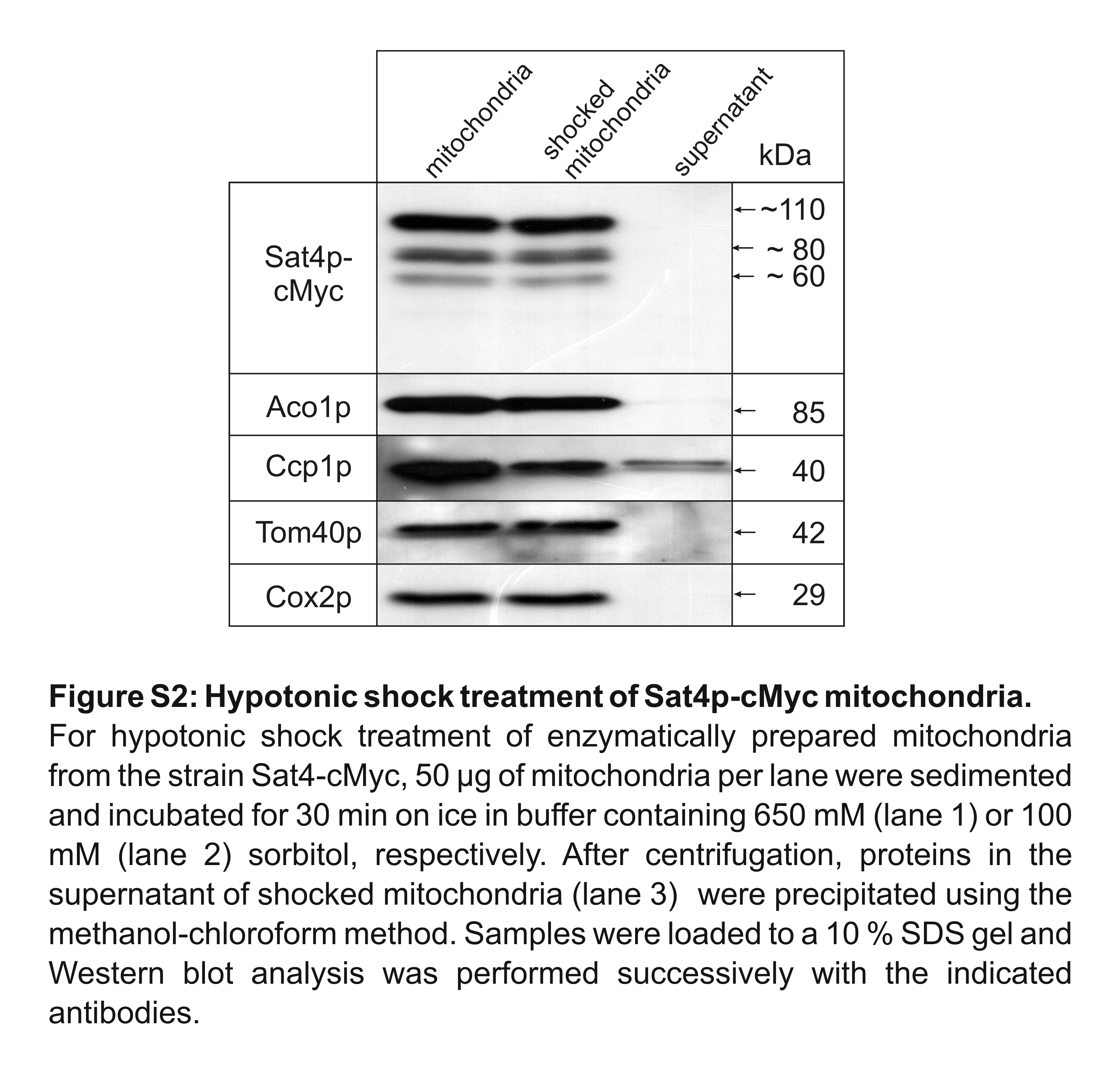

Supplement: Figure S2 — Hypotonic shock treatment of Sat4p-cMyc mitochondria. For hypotonic shock treatment of enzymatically prepared mitochondria from the strain Sat4-cMyc, 50 µg of mitochondria per lane were sedimented and incubated for 30 min on ice in buffer containing 650 mM (lane 1) or 100 mM (lane 2) sorbitol, respectively. After centrifugation, proteins in the supernatant of shocked mitochondria (lane 3) were precipitated using the methanol-chloroform method. Samples were loaded to a 10% SDS gel and Western blot analysis was performed successively with the indicated antibodies. (TIF) [file pone.0103956.s002.tif]

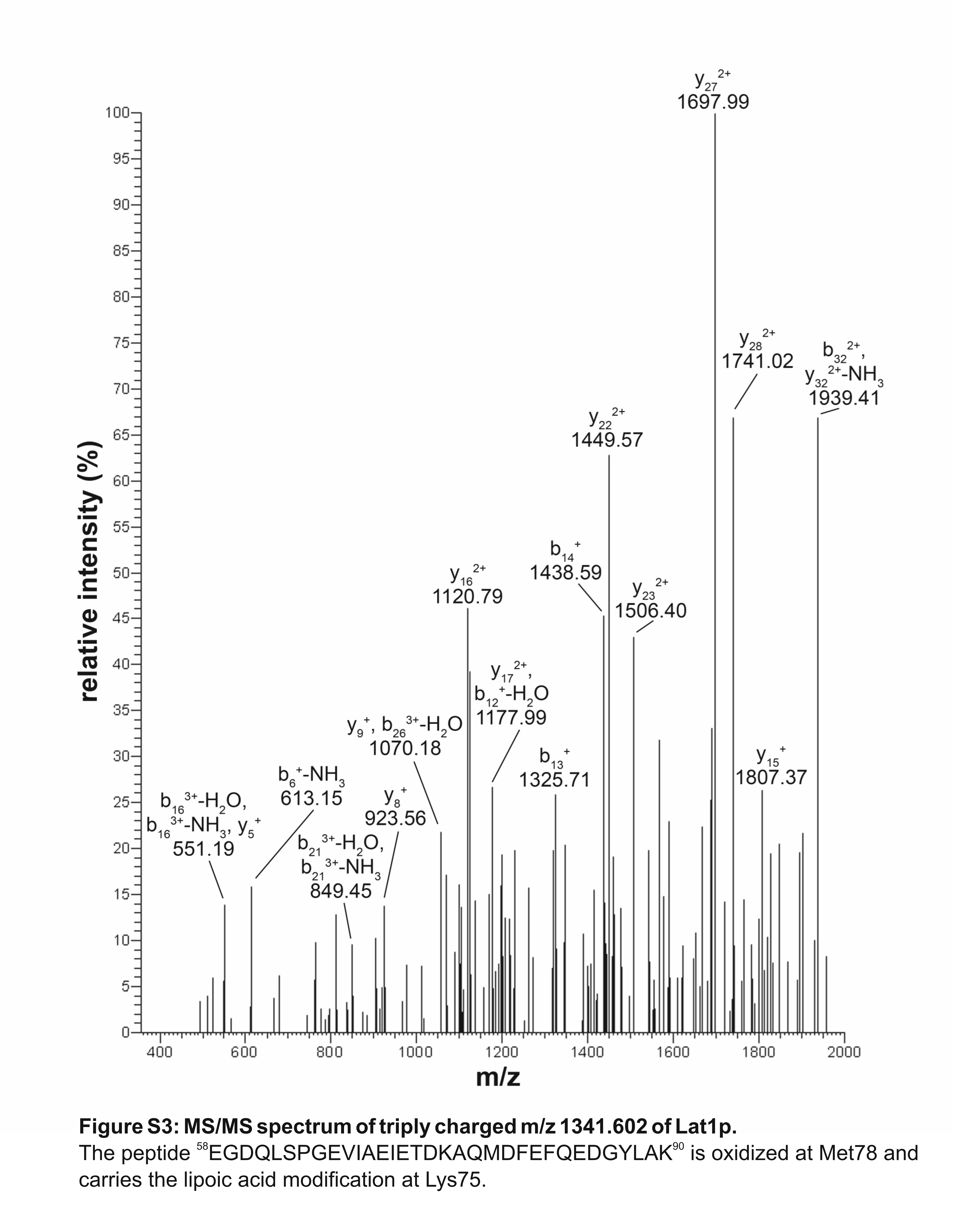

Supplement: Figure S3 — MS/MS spectrum of triply charged m/z 1341.602 of Lat1p. The peptide 58EGDQLSPGEVIAEIETDKAQMDFEFQEDGYLAK90 is oxidized at Met78 and carries the lipoic acid modification at Lys75. (TIF) [file pone.0103956.s003.tif]

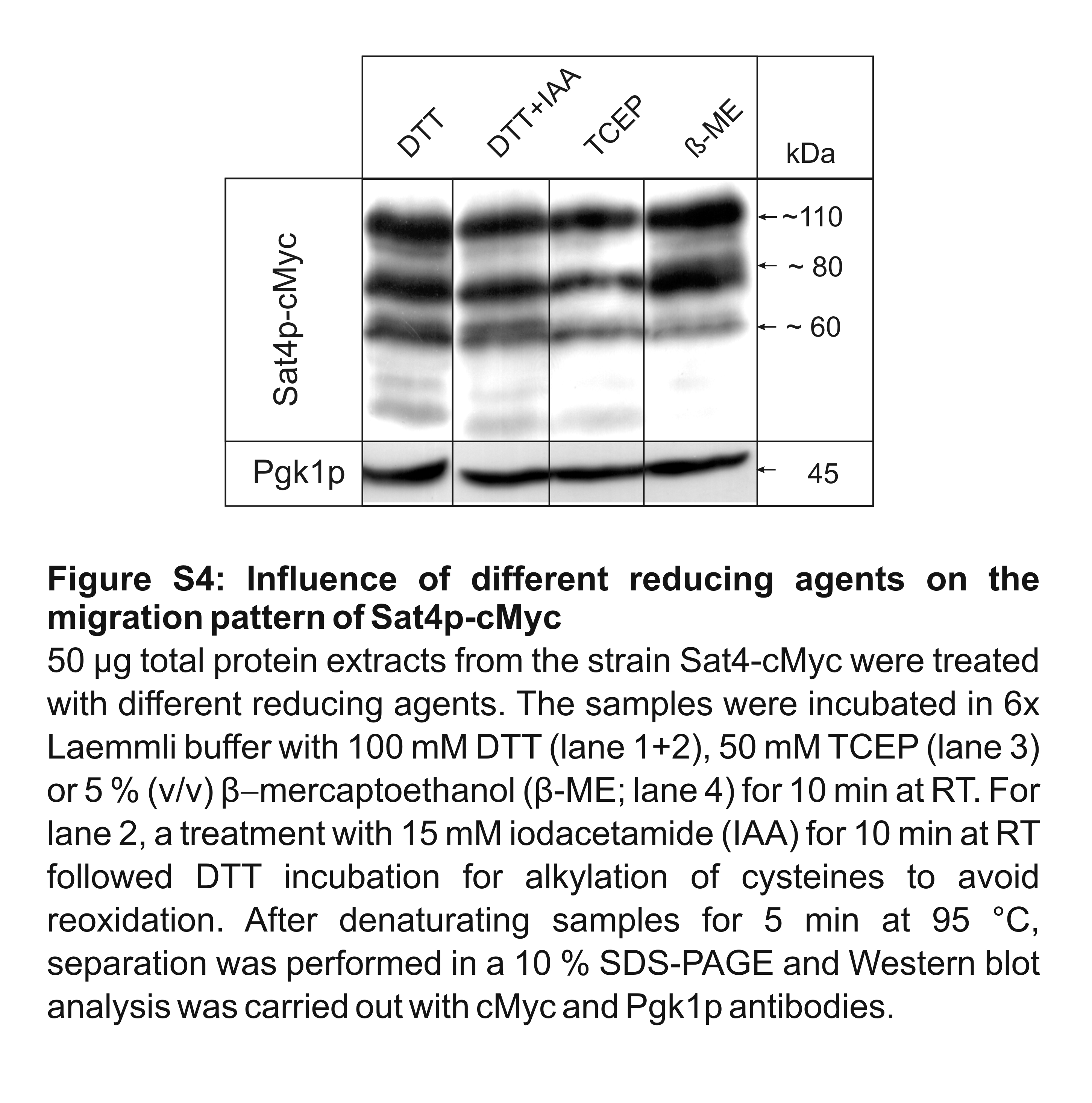

Supplement: Figure S4 — Influence of different reducing agents on the migration pattern of Sat4p-cMyc. 50 µg total protein extracts from the strain Sat4-cMyc were treated with different reducing agents. The samples were incubated in 6× Laemmli buffer with 100 mM DTT (lane 1+2), 50 mM TCEP (lane 3) or 5% (v/v) β-mercaptoethanol (β-ME; lane 4) for 10 min at RT. For lane 2, a treatment with 15 mM iodacetamide (IAA) for 10 min at RT followed DTT incubation for alkylation of cysteines to avoid reoxidation. After denaturating samples for 5 min at 95°C, separation was performed in a 10% SDS-PAGE and Western blot analysis was carried out with cMyc and Pgk1p antibodies. (TIF) [file pone.0103956.s004.tif]
